# Treatment pattern and clinical outcomes of remdesivir in hospitalized COVID-19 patients with severe chronic kidney disease: a database analysis of acute care hospitals in Japan

**DOI:** 10.1007/s10157-024-02609-0

**Published:** 2024-12-30

**Authors:** Manami Yoshida, Nao Taguchi, Yi Piao, Rikisha Gupta, Mark Berry, Jami Peters, Mazin Abdelghany, Mel Chiang, Chen-Yu Wang, Hiroshi Yotsuyanagi

**Affiliations:** 1 Gilead Sciences, K.K., 16/F GRAN TOKYO SOUTH TOWER, 1-9-2, Marunouchi, Chiyoda-ku, Tokyo, 100-6616 Japan; 2 Gilead Sciences, Inc, 333 Lakeside Dr, Foster City, CA USA; 3 The Institute of Medical Science, The University of Tokyo, Tokyo, Japan

**Keywords:** COVID-19, Chronic kidney disease, Hospitalization, Mortality, Disease progression, Remdesivir

## Abstract

**Background:**

There is limited evidence on clinical outcomes and treatment pattern in Japanese patients with severe chronic kidney disease (CKD), hospitalized for coronavirus disease-2019 (COVID-19). We aimed to describe patient characteristics, treatment pattern, and clinical outcomes in Japanese patients with severe CKD, hospitalized for COVID-19 who received remdesivir (RDV).

**Methods:**

We used the anonymized claims database from Medical Data Vision Co., Ltd., Japan. The analysis included patients aged ≥ 18 years with severe CKD, hospitalized for moderate to severe COVID-19, and administered ≥ 1 dose of RDV between October 2021 and September 2023. All-cause inpatient mortality, disease progression, and recovery up to 56 days from hospitalization were evaluated.

**Results:**

Data of 847 patients were analyzed (mean age 73.0 ± 14.1 years). Median (Q1–Q3) time to RDV initiation was 1.0 day (1.0–2.0) from hospitalization and treatment duration was 5.0 days (3.0–5.0). At RDV initiation, 44.27% patients required non-invasive positive pressure ventilation/high or low flow oxygen; 4.25% required invasive mechanical ventilation/extracorporeal membrane oxygenation/intensive care unit hospitalization. Proportion of patients with all-cause mortality was 11.45% (stage 4, 14.89%; stage 5, 10.47%) by 28 days and 12.28% (stage 4, 16.49%; stage 5, 11.08%) by 56 days. At 28 days, 12.28% had disease progression and 72.14% recovered.

**Conclusion:**

Most patients with severe CKD received RDV immediately after hospitalization. The majority of patients recovered by 28 days. The study provided insights into RDV treatment in inpatient settings, which could contribute to the discussion on standard of care in this population in Japan.

**Supplementary Information:**

The online version contains supplementary material available at 10.1007/s10157-024-02609-0.

## Introduction

The burden of coronavirus disease-2019 (COVID-19) continued to rise from early 2020 in Japan [1]. Globally, vaccination has reduced the risk of severe illness or death [2]. Nevertheless, the risk of disease progression remains for patients who are older, immunocompromised, and/or have chronic diseases such as diabetes or chronic kidney disease (CKD) [3, 4].

Patients with concomitant COVID-19 and CKD have a high risk of severe illness, requiring intensive care unit (ICU) admission and invasive mechanical ventilation (IMV), or death [5–7]. European registry data showed that 28-day mortality risk was 21.1 times in dialysis patients with COVID-19 and mortality attributable to COVID-19 was 20.0% vs 1.2% without COVID-19 [8]. In Japan, nationwide studies demonstrated that patients with CKD and COVID-19 require oxygen support [9] and older patients with COVID-19 (aged ≥ 70 years) undergoing dialysis have a higher risk of mortality than the general population [10] and those aged < 60 years [11].

According to COVID-19 treatment guidelines, therapies targeting severe acute respiratory syndrome coronavirus-2 (SARS-CoV-2) are anticipated to have greatest effect early in the disease course [12]. Remdesivir (RDV), a nucleoside analogue SARS-CoV-2 RNA-dependent RNA polymerase inhibitor, is recommended for treating patients with mild to moderate COVID-19 at high risk of progressing to severe COVID-19 [12]. In Japan, RDV received emergency approval for treating COVID-19 in May 2020 [13] and is recommended in the treatment guidelines [14, 15] regardless of the disease severity.

Patients with severe CKD were excluded from some clinical trials evaluating the efficacy and safety of RDV in treatment of COVID-19 [16–18]. The REDPINE study demonstrated that RDV dosed once daily up to day 5 was well tolerated in patients with moderately and severely reduced kidney function and hospitalized for COVID-19 [19]. A nationwide cohort study of Japanese patients undergoing dialysis showed significantly lower mortality risk (hazard ratio 0.60, 95% confidence interval [CI] 0.37–0.98, *p* = 0.041) in patients with RDV treatment vs no RDV treatment [11]. A single-center retrospective study in Japan showed safety of RDV in patients with severe CKD and reported no differences in the 30-day mortality between patients with estimated glomerular filtration rate (eGFR) ≤ 30 mL/min and those with eGFR > 30 mL/min (relative risk 1.00; 95% CI 0.18–5.56) [20].

Here, we aimed to describe patient characteristics and treatment pattern, and to evaluate clinical outcomes in patients with severe CKD, hospitalized with COVID-19 and treated with RDV in Japan, using a large-scale database.

## Materials and methods

### Study design and data source

A single-arm, retrospective, non-interventional, cohort study was conducted using anonymized claims data from Medical Data Vision (MDV) Co., Ltd., Japan. The MDV database, as of January 2024, includes data from > 480 hospitals and 46.27 million patients of all ages in Japan [21, 22]. The study period was from 19 October 2020 to 30 September 2023. The index date was the date of RDV treatment initiation. The baseline period was up to 12 months prior to the index date. Patients were followed for up to 56 days from the index to in-hospital death, lost to follow-up, or end of study follow-up, whichever occurred earlier.

### Study participants

Patients had to be ≥ 18 years at the index and have ≥ 1 International Classification of Diseases, 10th revision (ICD-10) diagnosis code for CKD stage 4 or 5, or dialysis, or measurement of eGFR < 30 mL/min/1.73 m^2^ during the baseline period. The detailed codes to define CKD stages 4 and 5, or dialysis are provided in Supplementary Table S1. Only patients hospitalized with moderate I, II, or severe COVID-19 (ICD-10 code: B342, U0.7.X-U10.X1 as per their medical claims) who received ≥ 1 dose of RDV either alone or in conjunction with other medications during hospitalization (based on Anatomical Therapeutic Chemical code: J05AB16) between 18 October 2021 and 30 September 2023 were included. Severity of COVID-19 at index was defined as moderate I (patients having a record of Emergency Medical Management for moderate COVID-19 and not meeting the criteria for moderate II or severe); moderate II (patients requiring non-invasive positive pressure ventilation [NPPV], high flow oxygen, or low flow oxygen), and severe (patients requiring ICU hospitalization, extracorporeal membrane oxygenation [ECMO], or IMV). Subgroups of CKD stage 4 were defined as those with ICD-10 code N184 or 15 ≤ eGFR < 30 mL/min/1.73 m^2^, excluding those who met the criteria for CKD stage 5; and CKD stage 5 defined as those with ICD-10 code N185 or eGFR < 15 mL/min/1.73 m^2^ or dialysis at index.

Patients were excluded if they had evidence of prior COVID-19 inpatient hospitalization in the baseline period. Patients were also excluded if sex or year of birth was missing from their record during the baseline period.

### Study endpoints

Primary endpoint was inpatient all-cause mortality by 28 days post-index. Secondary endpoints were: all-cause mortality through 7 and 14 days; rate of disease progression at 28 days in patients with moderate I or II COVID-19 at index (defined as having a record for ECMO/IMV or ICU hospitalization, or as death during follow-up) and in patients with severe COVID-19 (ECMO/IMV) at index (defined as death during follow-up); rate of recovery from COVID-19 at discharge (patients with the status of cured or improved as reason for discharge) by 28 days; treatment pattern of RDV including time to RDV initiation after hospital admission (days); duration of RDV treatment (days; from index to final RDV prescription date); duration of ICU admission (days); and concomitant medication (corticosteroid, baricitinib, tocilizumab, or heparin) during RDV prescription period. Furthermore, exploratory endpoints were inpatient all-cause mortality, rate of disease progression, and rate of recovery from COVID-19 by 56 days post-index.

### Statistical analysis

Statistical analyses were performed using SAS, version 9.4 (SAS Institute Inc., Cary, NC, USA). Categorical variables were reported as numbers (n) and percentages (%) of patients with 95% CIs. Continuous variables were reported as mean, standard deviation (SD), first and third quartiles (Q1, Q3), median, and 95% CIs, with total number of observations and number of missing values.

All-cause inpatient mortality and disease progression were estimated using Kaplan–Meier (KM) curves and reported as proportion of patients with 95% CIs. Patients were followed for the defined observation period unless they experienced the event of interest (death) or were censored due to lost to follow-up. The Clopper-Pearson method calculated the 95% CIs for percentage of patients with all-cause mortality, disease progression, and COVID-19 recovery, with all patients in the analysis as the denominator.

Study outcomes were evaluated in the subgroups of CKD stages 4 and 5, COVID-19 severity (moderate I, moderate II, severe), and age (18–49 years, 50–64 years, and ≥ 65 years) at index. A subgroup analysis was performed on patients with eGFR < 30 mL/min/1.73 m^2^ during the baseline period. As laboratory data were available for only 10% of patients, those with CKD stage 4 or 5 diagnosis codes but no laboratory data were included. We explored whether significant difference existed between the overall population and eGFR < 30 mL/min/1.73 m^2^ subgroup, which is a more objective CKD stage measure.

### Ethics

In this study, data were deidentified prior to entry. As Japanese Ethical Guidelines for Medical and Health Research Involving Human Subjects do not apply to studies of secondary analyses using anonymized data, review of an institutional review board/research ethics committee was not required. No informed consent was obtained.

## Results

### Patient characteristics

**Fig. 1:**
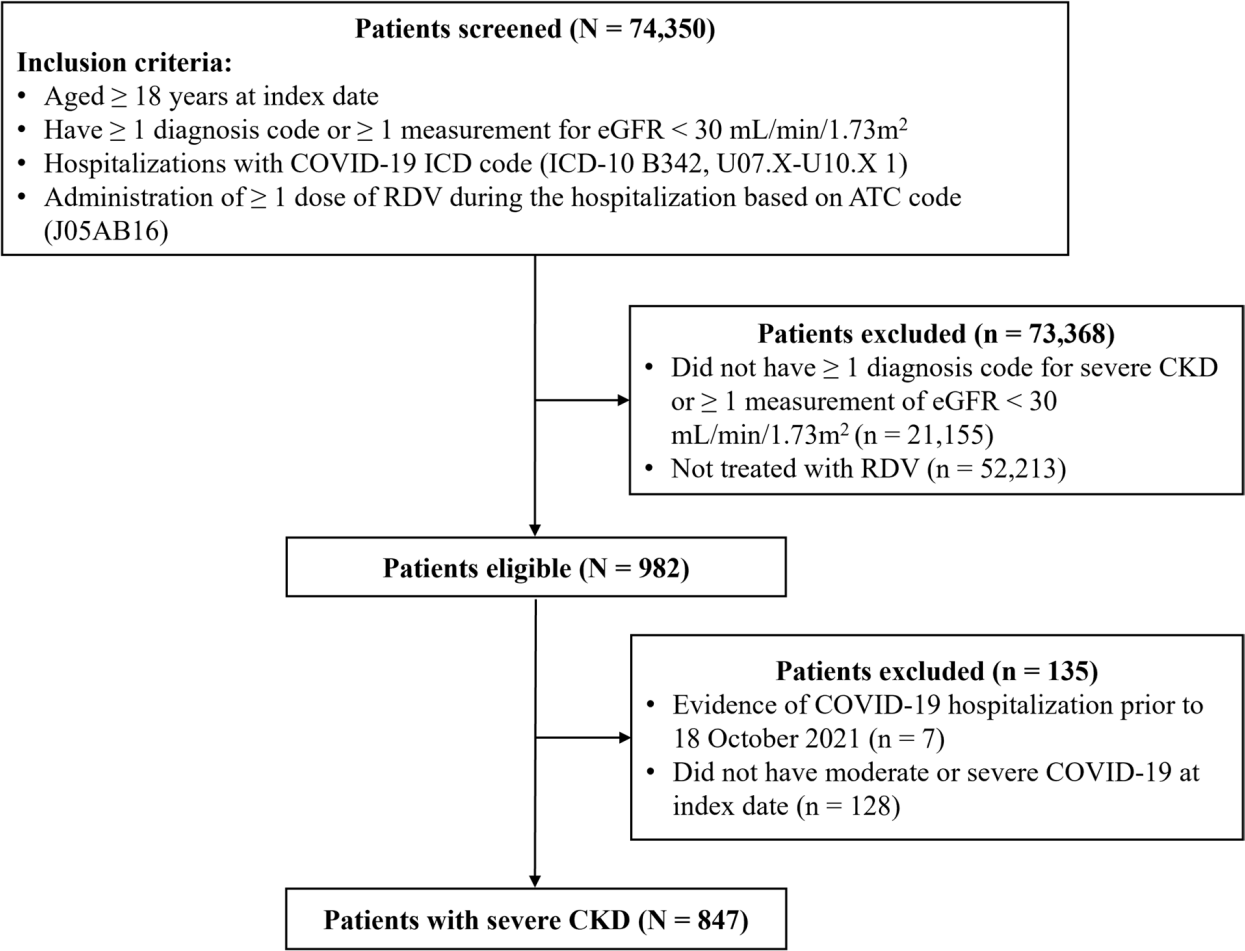
Patient selection flow chart. *ATC*, anatomical therapeutic chemical; *CKD*, chronic kidney disease; *COVID-19*, coronavirus disease-2019; *eGFR*, estimated glomerular filtration rate; *ICD*, International Classification of Diseases; *RDV*, remdesivir

Of 74,350 patients hospitalized for COVID-19 during the study period, 982 patients with severe CKD met the inclusion criteria. Of these, 135 patients were excluded due to hospitalization during baseline period for COVID-19 or absence of a record for moderate or severe COVID-19 at the index. Thus, 847 patients were included (Fig. 1).

Mean age (± SD) of severe CKD patients at index was 73.0 ± 14.1 years, with most patients aged ≥ 65 years (75.68%) and male (69.07%). Cardiovascular disease (93.98%) and hypertension (79.46%) were the most common comorbidities. At index, 51.48% patients had moderate I, 44.27% had moderate II, and 4.25% had severe COVID-19 (Table 1). Some patients with end-stage CKD at baseline were included; 67.53% were diagnosed with CKD stage 5, 13.81% had eGFR < 15 mL/min/1.73 m^2^, and 40.26% were undergoing dialysis (Table 1). These codes were not mutually exclusive: patients could have > 1 code for severe CKD.

**Table 1:** Demographic and clinical characteristics of patients with severe CKD

Characteristic	Patients with severe CKD (n = 847)	Patients with CKD stage 4 (n = 188)	Patients with CKD stage 5 (n = 659)	COVID-19 severity (at index) group			Age group (years)		
				Moderate I (n = 436)	Moderate II (n = 375)	Severe (n = 36)	18–49 (n = 58)	50–64 (n = 148)	≥ 65 (n = 641)
Age (years)									
Mean ± SD	73.0 ± 14.1	82.1 ± 11.0	70.4 ± 13.8	71.4 ± 15.1	75.0 ± 12.9	72.1 ± 12.2	41.5 ± 6.7	57.5 ± 4.4	79.5 ± 8.2
Age group (years)									
18–49	58 (6.85)	3 (1.60)	55 (8.35)	39 (8.94)	17 (4.53)	2 (5.56)	58 (100.00)	NA	NA
50–64	148 (17.47)	9 (4.79)	139 (21.09)	85 (19.50)	56 (14.93)	7 (19.44)	NA	148 (100.00)	NA
≥ 65	641 (75.68)	176 (93.62)	465 (70.56)	312 (71.56)	302 (80.53)	27 (75.00)	NA	NA	641 (100.00)
Sex									
Male	585 (69.07)	110 (58.51)	475 (72.08)	302 (69.27)	254 (67.73)	29 (80.56)	44 (75.86)	110 (74.32)	431 (67.24)
Female	262 (30.93)	78 (41.49)	184 (27.92)	134 (30.73)	121 (32.27)	7 (19.44)	14 (24.14)	38 (25.68)	210 (32.76)
Comorbidities									
COPD	110 (12.99)	41 (21.81)	69 (10.47)	49 (11.24)	52 (13.87)	9 (25.00)	5 (8.62)	13 (8.78)	92 (14.35)
Cardiovascular disease	796 (93.98)	176 (93.62)	620 (94.08)	408 (93.58)	356 (94.93)	32 (88.89)	50 (86.21)	137 (92.57)	609 (95.01)
Hypertension	673 (79.46)	148 (78.72)	525 (79.67)	355 (81.42)	293 (78.13)	25 (69.44)	43 (74.14)	114 (77.03)	516 (80.50)
Diabetes mellitus	512 (60.45)	102 (54.26)	410 (62.22)	246 (56.42)	242 (64.53)	24 (66.67)	29 (50.00)	98 (66.22)	385 (60.06)
ESCKD									
Dialysis	341 (40.26)	NA	341 (51.75)	191 (43.81)	131 (34.93)	19 (52.78)	22 (37.93)	69 (46.62)	250 (39.00)
CKD stage 5 (with ICD-10 code: N185)	572 (67.53)	NA	572 (86.80)	327 (75.00)	230 (61.33)	15 (41.67)	48 (82.76)	122 (82.43)	402 (62.71)
eGFR < 15 mL/min/1.73 m^2^	117 (13.81)	NA	117 (17.75)	66 (15.14)	47 (12.53)	4 (11.11)	8 (13.79)	20 (13.51)	89 (13.88)
COVID-19 disease severity at index									
Moderate I	436 (51.48)	75 (39.89)	361 (54.78)	436 (100.00)	NA	NA	2 (3.45)	7 (4.73)	27 (4.21)
Moderate II	375 (44.27)	107 (56.91)	268 (40.67)	NA	375 (100.00)	NA	17 (29.31)	56 (37.84)	302 (47.11)
Severe	36 (4.25)	6 (3.19)	30 (4.55)	NA	NA	36 (100.00)	39 (67.24)	85 (57.43)	312 (48.67)

All data are presented as mean ± SD or n (%) unless otherwise specified

Subgroups of CKD stage 4 defined as those with ICD-10 code N184 or 15 ≤ eGFR < 30 mL/min/1.73 m^2^, excluding those who met the criteria for CKD stage 5; and CKD stage 5 defined as those with ICD-10 code N185 or eGFR < 15 mL/min/1.73 m^2^ or dialysis at index

COVID-19 severity defined as *moderate I,* patients with a record of Emergency Medical Management for moderate COVID-19 and not meeting the criteria for moderate II or severe; *moderate II,* patients requiring non-invasive positive pressure ventilation, high flow oxygen or low flow oxygen; and *severe,* patients requiring invasive mechanical ventilation/extracorporeal membrane oxygenation, or intensive care unit hospitalization

*CKD*, chronic kidney disease; *COPD*, chronic obstructive pulmonary disease; *COVID-19,* coronavirus disease-2019; *eGFR*, estimated glomerular filtration rate; *ESCKD*, end-stage chronic kidney disease; *ICD-10*, International Classification of Diseases, 10th revision; *NA*, not applicable; *SD*, standard deviation

Of 847 patients, 188 had CKD stage 4 and 659 had CKD stage 5. Subgroups with CKD stage 4 (mean ± SD age, 82.1 ± 11.0 years) were about 10 years older than those with CKD stage 5 (70.4 ± 13.8 years). The proportion of patients with moderate I COVID-19 was less (39.89%) in CKD stage 4 subgroup than in CKD stage 5 (54.78%). Contrastingly, the proportion with moderate II COVID-19 was higher (56.91%) in CKD stage 4 than stage 5 40.67%). Patient characteristics were mostly similar in age and COVID-19 severity groups at index. More than 50% patients in severe COVID-19 had end-stage CKD undergoing dialysis (Table 1).

Clinical characteristics of patients with eGFR < 30 mL/min/1.73 m^2^ identified based on the assessment during baseline period (n = 253) (Supplementary Table S2) were similar to those observed in overall patients with severe CKD (n = 847, defined by diagnosis codes in the claims data; Table 1).

### Treatment pattern

Median time (Q1–Q3) to RDV initiation from hospital admission was 1.0 (1.0–2.0) day (except severe COVID-19 patients, 2.0 [1.0–3.0] days) and median duration (Q1–Q3) of RDV treatment was 5.0 (3.0–5.0) days in patients with severe CKD and similar among CKD stage 4 and 5 groups. Median duration of ICU admission (Q1–Q3) was 7.0 (3.0–12.0) days in patients with severe CKD (5.0 [1.0–13.0] days in stage 4 and 7.5 [3.5–12.0] days in stage 5). Patients received heparin (42.15%), corticosteroid (38.13%), baricitinib (1.89%), and tocilizumab (1.53%) concomitantly with RDV (Table 2). The RDV treatment pattern in patients with eGFR < 30 mL/min/1.73 m^2^ was similar to that in patients with severe CKD (Supplementary Table S3).

**Table 2:** Treatment pattern of remdesivir in patients with severe CKD

Treatment pattern	Patients with severe CKD	Patients with CKD stage 4	Patients with CKD stage 5	COVID-19 severity (at index) group			Age group (years)		
				Moderate I	Moderate II	Severe	18–49	50–64	≥ 65
Duration of remdesivir treatment (days)									
n	847	188	659	436	375	36	58	148	641
Mean ± SD	4.6 ± 2.2	4.9 ± 2.3	4.6 ± 2.1	4.5 ± 2.1	4.8 ± 2.1	5.0 ± 3.0	4.8 ± 2.5	4.5 ± 1.8	4.7 ± 2.2
Median (Q1–Q3)	5.0 (3.0–5.0)	5.0 (3.0–5.0)	5.0 (3.0–5.0)	5.0 (3.0–5.0)	5.0 (4.0–5.0)	5.0 (2.5–6.5)	5.0 (3.0–6.0)	5.0 (3.0–5.0)	5.0 (3.0–5.0)
Time to remdesivir initiation from hospital admission (days)									
n	766	156	610	394	342	30	57	146	563
Mean ± SD	1.9 ± 2.5	2.1 ± 2.9	1.9 ± 2.4	1.7 ± 1.5	2.1 ± 3.2	3.2 ± 3.0	1.8 ± 2.1	2.0 ± 1.8	1.9 ± 2.7
Median (Q1–Q3)	1.0 (1.0–2.0)	1.0 (1.0–2.0)	1.0 (1.0–2.0)	1.0 (1.0–2.0)	1.0 (1.0–2.0)	2.0 (1.0–3.0)	1.0 (1.0–1.0)	1.0 (1.0–2.0)	1.0 (1.0–2.0)
Duration of ICU admission^a^ (days)									
n	19	3	16	0	1	18	1	3	15
Mean ± SD	8.0 ± 6.1	6.3 ± 6.1	8.3 ± 6.3	–	1.0 (–)	8.4 ± 6.1	21.0 (–)	9.7 ± 4.2	6.8 ± 5.6
Median (Q1–Q3)	7.0 (3.0–12.0)	5.0 (1.0–13.0)	7.5 (3.5–12.0)	–	1.0 (1.0–1.0)	7.5 (4.0–12.0)	21.0 (21.0–21.0)	11.0 (5.0–13.0)	5.0 (2.0–12.0)
Concomitant drugs^b^									
n	847	188	659	436	375	36	58	148	641
Corticosteroid	323 (38.13)	89 (47.34)	234 (35.51)	85 (19.50)	210 (56.00)	28 (77.78)	26 (44.83)	58 (39.19)	239 (37.29)
Baricitinib	16 (1.89)	15 (7.98)	1 (0.15)	0 (0.00)	15 (4.00)	1 (2.78)	0 (0.00)	0 (0.00)	16 (2.50)
Tocilizumab	13 (1.53)	4 (2.13)	9 (1.37)	0 (0.00)	10 (2.67)	3 (8.33)	2 (3.45)	1 (0.68)	10 (1.56)
Heparin	357 (42.15)	81 (43.09)	276 (41.88)	137 (31.42)	190 (50.67)	30 (83.33)	17 (29.31)	60 (40.54)	280 (43.68)

All data are presented as mean ± SD or n (%) unless otherwise specified

^a^Including patients who were admitted to the ICU during the follow-up period

^b^Includes data pertaining to COVID-19 related drugs

Subgroups of CKD stage 4 defined as those with ICD-10 code N184 or 15 ≤ eGFR < 30 mL/min/1.73 m^2^, excluding those who met the criteria for CKD stage 5; and CKD stage 5 defined as those with ICD-10 code N185 or eGFR < 15 mL/min/1.73 m^2^ or dialysis at index

COVID-19 severity defined as *moderate I,* patients with a record of Emergency Medical Management for moderate COVID-19 and not meeting the criteria for moderate II or severe; *moderate II,* patients requiring non-invasive positive pressure ventilation, high flow oxygen or low flow oxygen; and *severe,* patients requiring invasive mechanical ventilation/extracorporeal membrane oxygenation, or intensive care unit hospitalization

*CKD*, chronic kidney disease; *COVID-19,* coronavirus disease-2019; *eGFR*, estimated glomerular filtration rate; *ICD-10,* International Classification of Diseases, 10th revision; *ICU*, intensive care unit; *Q1*, first quartile; *Q3*, third quartile; *SD*, standard deviation

### Inpatient mortality

**Fig. 2:**
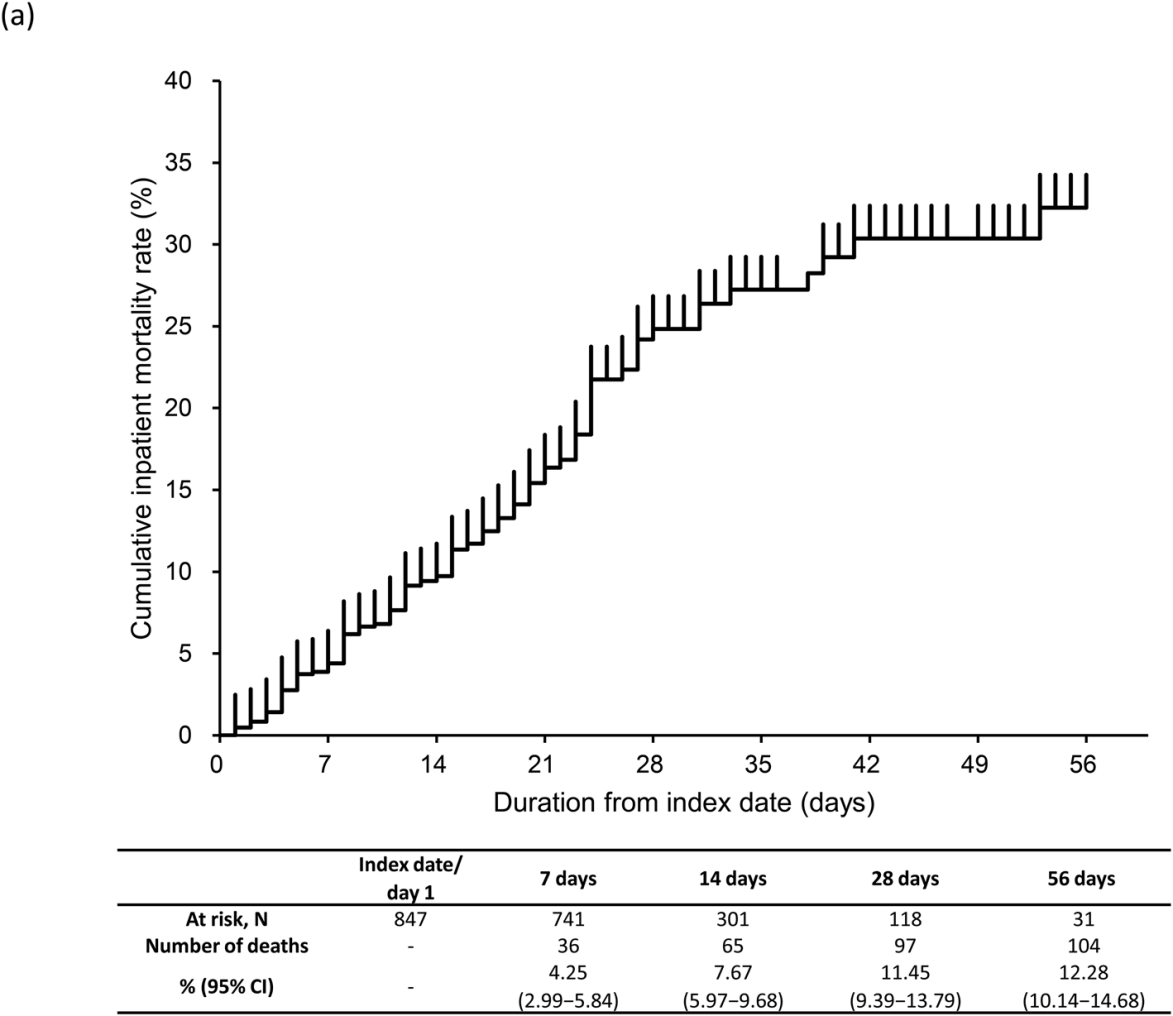

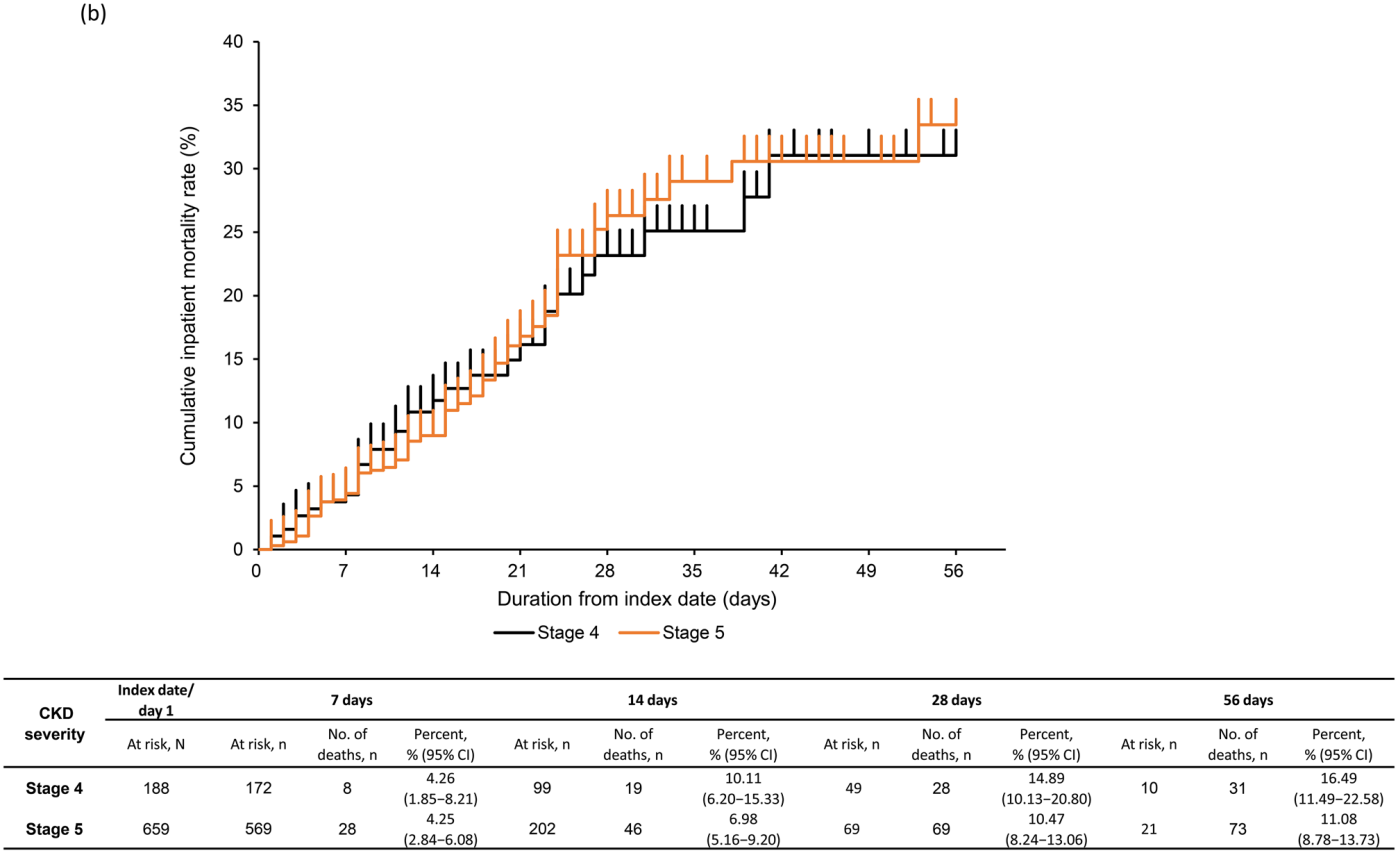

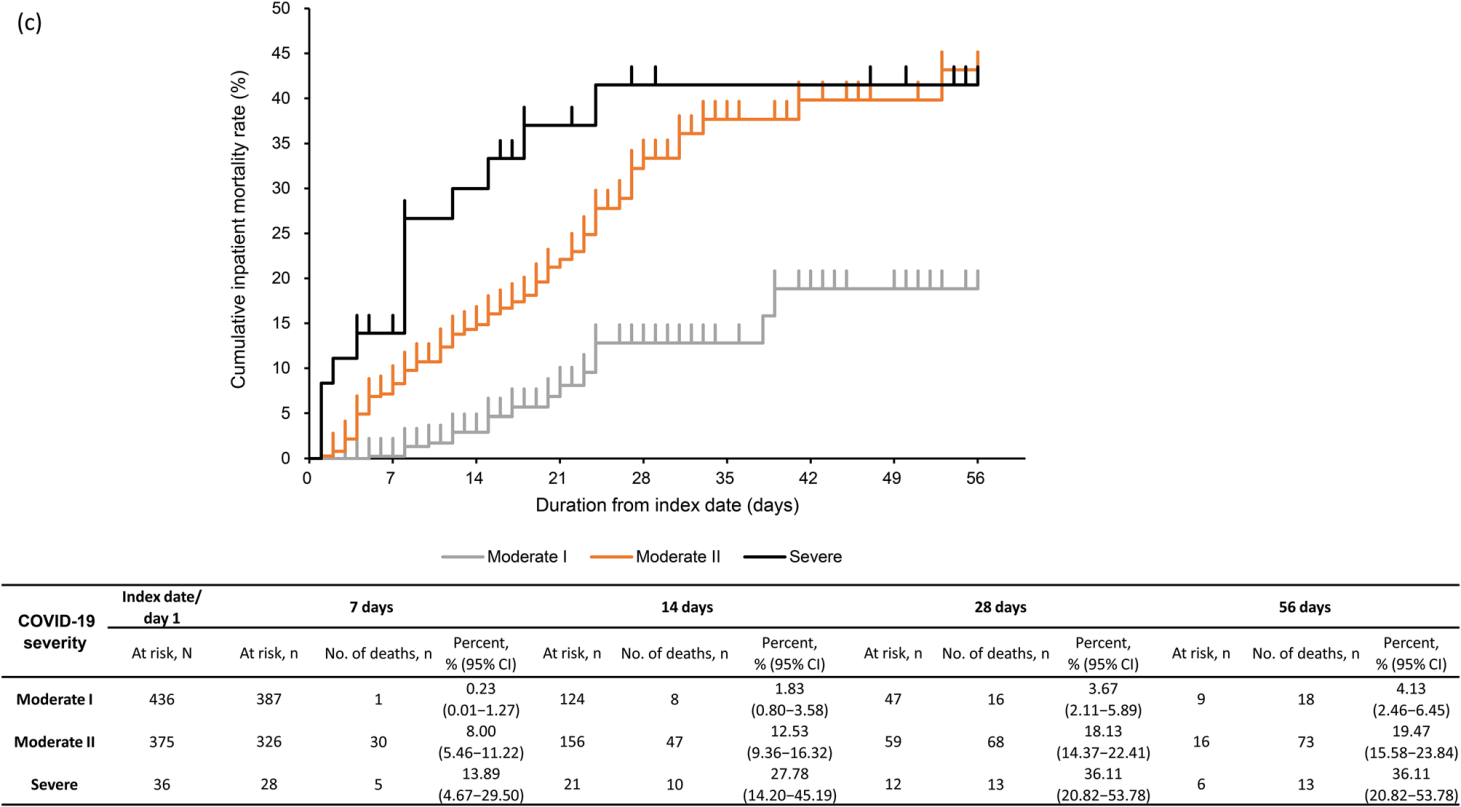

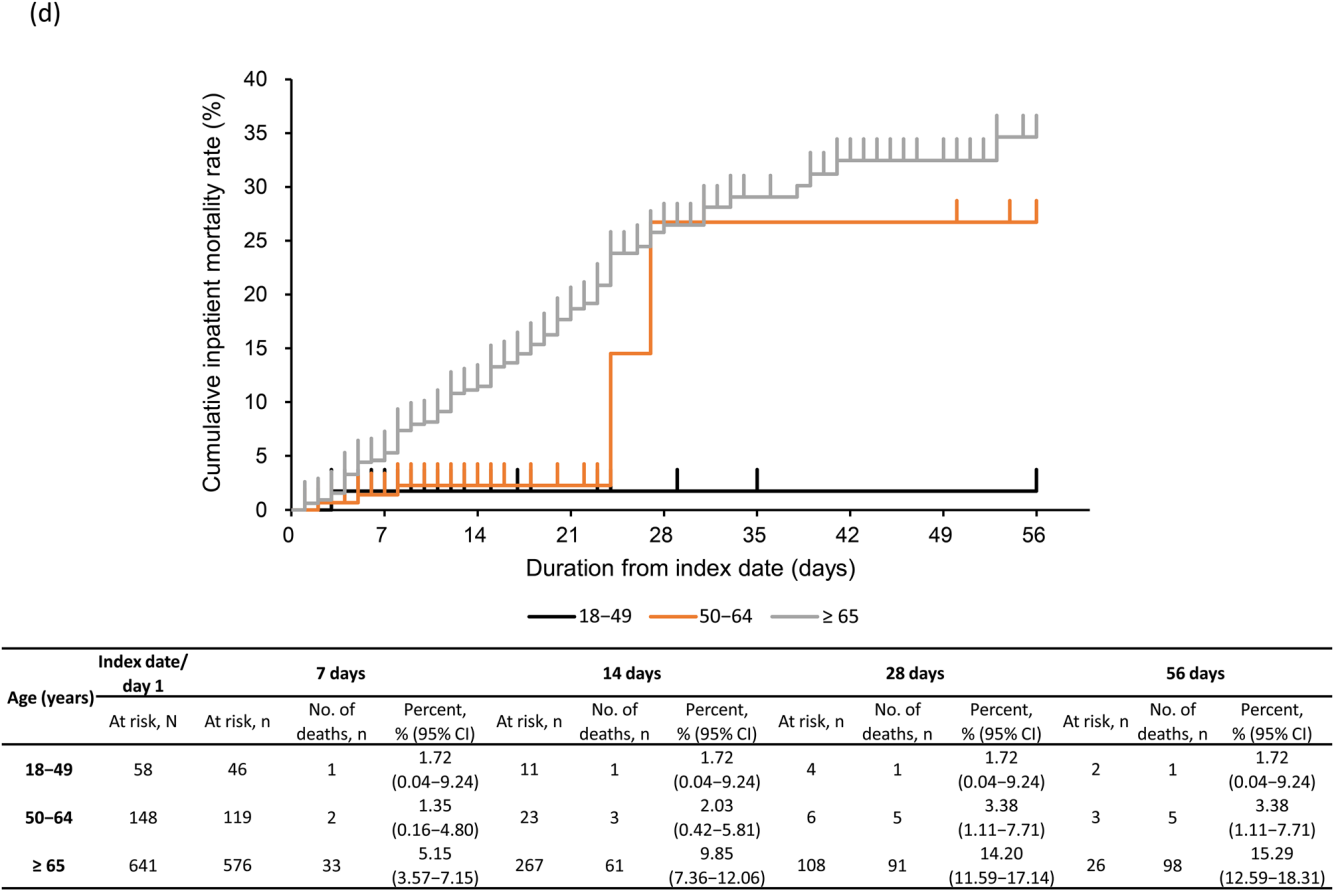
Kaplan–Meier curve of inpatient all-cause mortality through 56 days in **a** severe CKD patients, **b** severe CKD patients based on CKD stage, **c** severe CKD patients based on COVID-19 severity at index, **d** severe CKD patients based on age group. Subgroups of CKD stage 4 defined as those with ICD-10 code N184 or 15 ≤ eGFR < 30 mL/min/1.73 m^2^, excluding those who met the criteria for CKD stage 5; and CKD stage 5 defined as those with ICD-10 code N185 or eGFR < 15 mL/min/1.73 m^2^ or dialysis at index. COVID-19 severity defined as *moderate I,* patients with a record of Emergency Medical Management for moderate COVID-19 and not meeting the criteria for moderate II or severe; *moderate II,* patients requiring non-invasive positive pressure ventilation, high flow oxygen or low flow oxygen; and *severe,* patients requiring invasive mechanical ventilation/extracorporeal membrane oxygenation, or intensive care unit hospitalization. *CI*, confidence interval; *CKD*, chronic kidney disease; *COVID-19*, coronavirus disease-2019; *eGFR*, estimated glomerular filtration rate; *ICD-10*, International Classification of Diseases, 10th revision; *No.*, number

The KM curve for inpatient all-cause mortality is shown in Fig. 2a–d. The proportion of patients with inpatient all-cause mortality (95% CI) with severe CKD was 11.45% (9.39–13.79%) by 28 days and 12.28% (10.14–14.68%) by 56 days post-index (Fig. 2a). Inpatient all-cause mortality rate at 28 days was 14.89% (10.13–20.80%) and 10.47% (8.24–13.06%) in CKD stage 4 and 5 subgroups (Fig. 2b). By 28 days, inpatient all-cause mortality rate was 3.67% (2.11–5.89%) in patients with moderate I, 18.13% (14.37–22.41%) in patients with moderate II, and 36.11% (20.82–53.78%) in patients with severe COVID-19 (Fig. 2c). Inpatient all-cause mortality rate by 28 days in patients aged ≥ 65 years (14.20% [11.59–17.14%]) and 50–64 years (3.38% [1.11–7.71%]) was higher than in those aged 18–49 years (1.72% [0.04–9.24%]) (Fig. 2d). Inpatient all-cause mortality rates in patients with eGFR < 30 mL/min/1.73 m^2^ and by COVID-19 severity and age groups were similar (Supplementary Fig. S1a–c).

### Disease progression

**Fig. 3:**
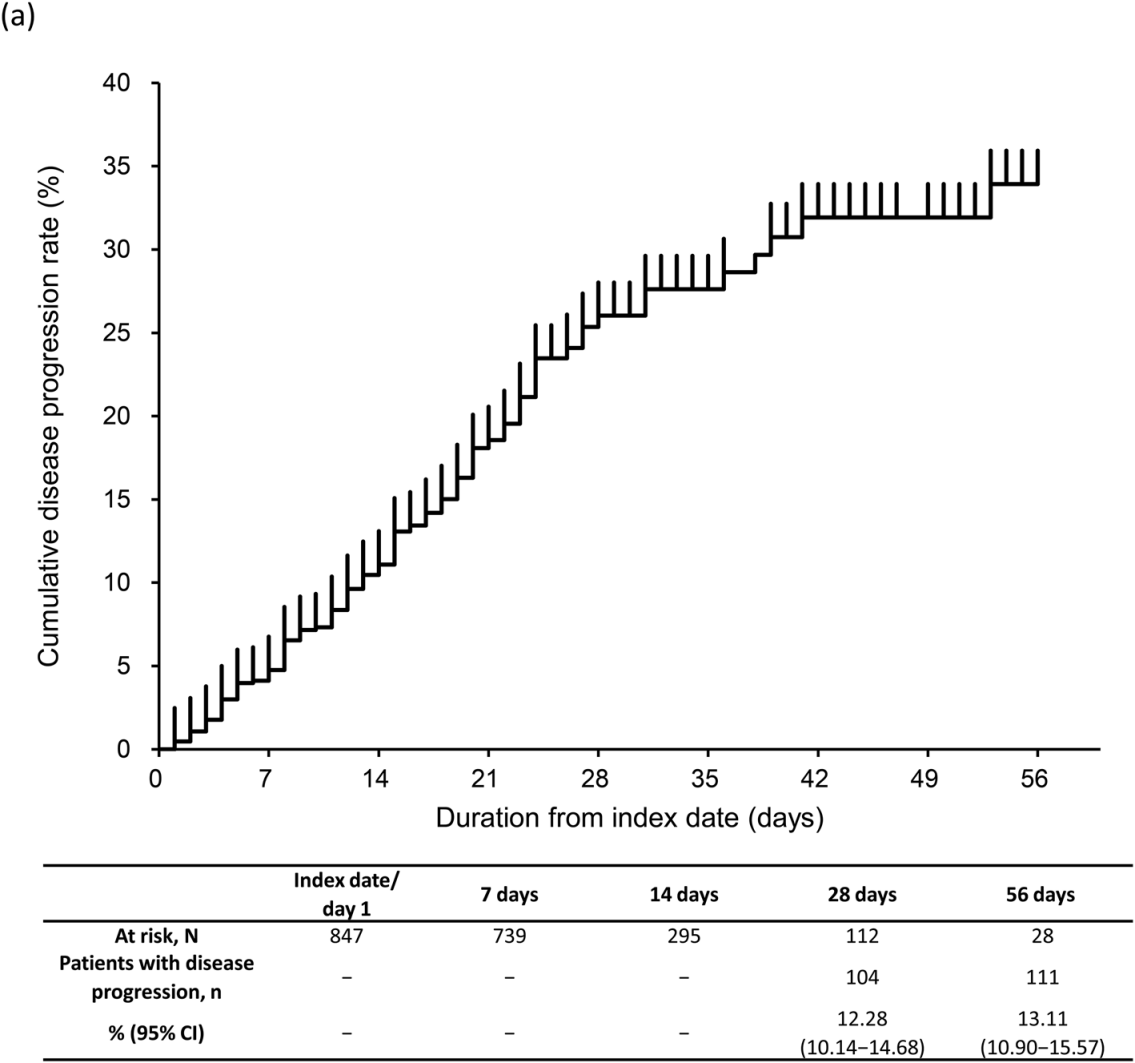

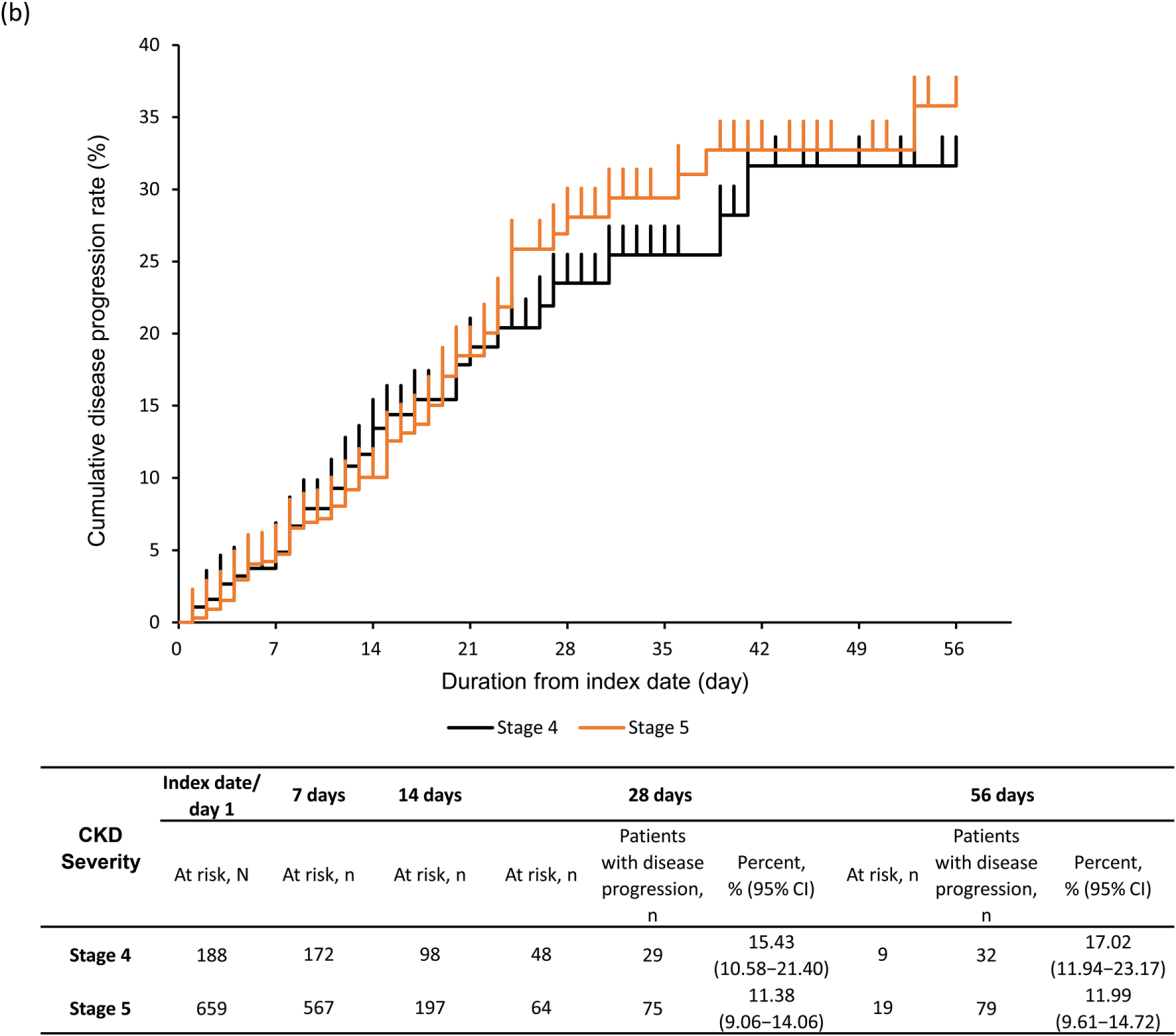

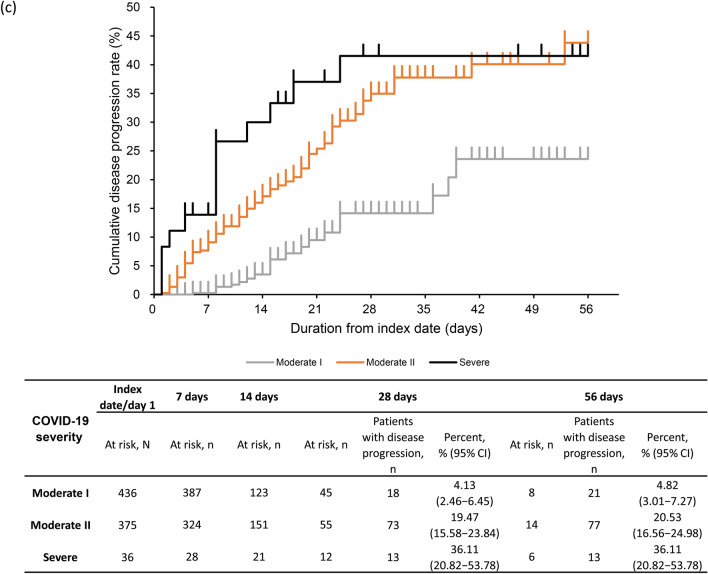

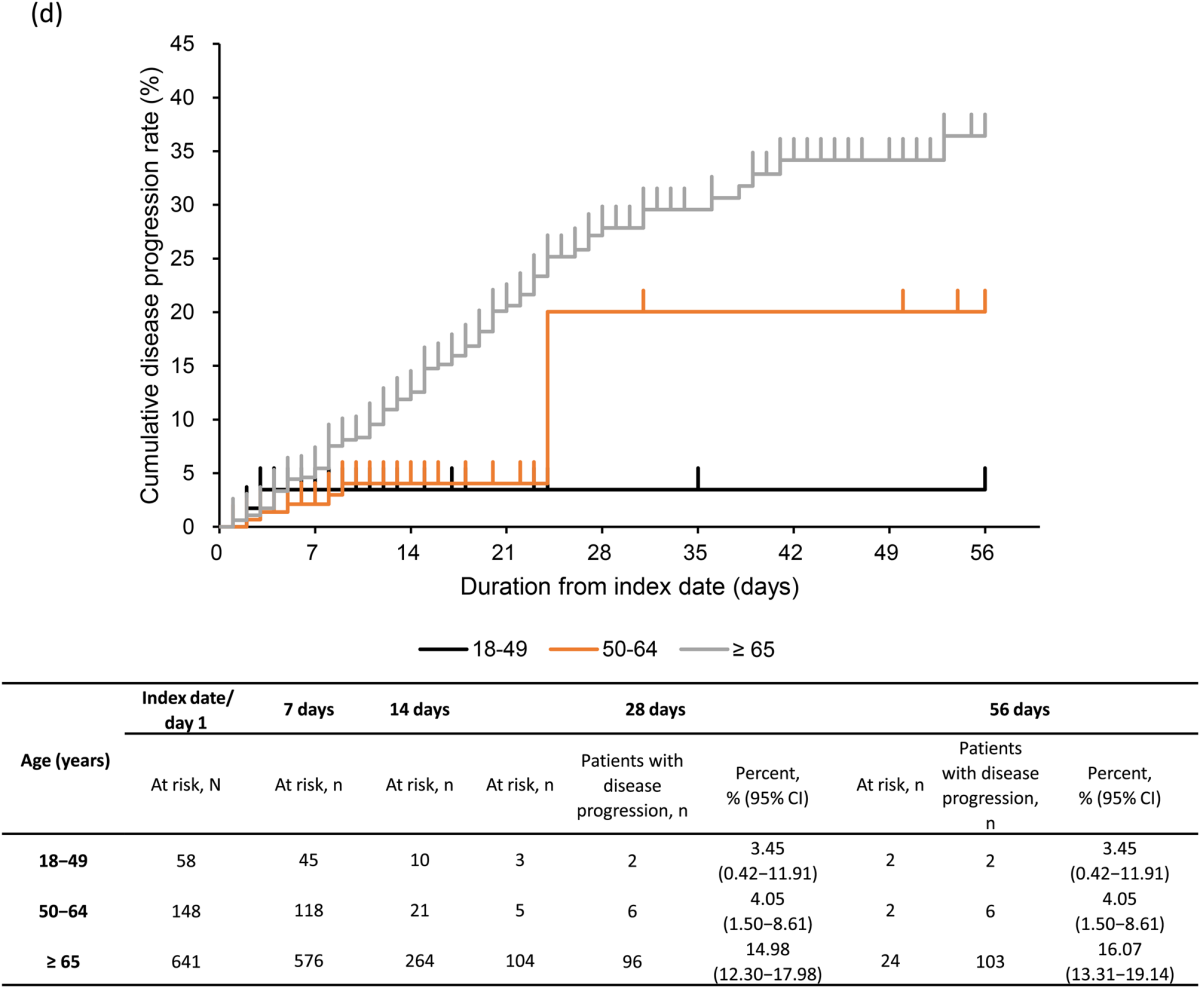
Kaplan–Meier curve for disease progression through 56 days in **a** severe CKD patients, **b** severe CKD patients based on CKD stage, **c** severe CKD patients based on COVID-19 severity at index, **d** severe CKD patients based on age group. Number and percentage of patients with disease progression estimated at 28 and 56 days. Disease progression in patients with moderate I or II COVID-19 at index, defined as having a record for invasive mechanical ventilation/extracorporeal membrane oxygenation or intensive care unit hospitalization, or as death during follow-up; and in patients with severe COVID-19 at index, defined as death during follow-up. Subgroups of CKD stage 4 defined as those with ICD-10 code N184 or 15 ≤ eGFR < 30 mL/min/1.73 m^2^, excluding those who met the criteria for CKD stage 5; and CKD stage 5 defined as those with ICD-10 code N185 or eGFR < 15 mL/min/1.73 m^2^ or dialysis at index. COVID-19 severity defined as *moderate I,* patients with a record of Emergency Medical Management for moderate COVID-19 and not meeting the criteria for moderate II or severe; *moderate II,* patients requiring non-invasive positive pressure ventilation, high flow oxygen or low flow oxygen; and *severe,* patients requiring invasive mechanical ventilation/extracorporeal membrane oxygenation, or intensive care unit hospitalization. *CI*, confidence interval; *CKD*, chronic kidney disease; *COVID-19*, coronavirus disease-2019; *eGFR*, estimated glomerular filtration rate; *ICD-10*, International Classification of Diseases, 10th revision

The rate of disease progression (95% CI) was 12.28% (10.14–14.68%) at 28 days and 13.11% (10.90–15.57%) at 56 days (Fig. 3a). At 28 days, the rate of disease progression was 15.43% (10.58–21.40%) and 11.38% (9.06–14.06%) in CKD stage 4 and 5 subgroups (Fig. 3b). The proportion of severe CKD patients with disease progression at 28 days was 4.13% (2.46–6.45%) in patients with moderate I, 19.47% (15.58–23.84%) in patients with moderate II, and 36.11% (20.82–53.78%) in patients with severe COVID-19 (Fig. 3c). At 28 days, the rate of disease progression was high, 14.98% (12.30–17.98%) in ≥ 65 years group vs 4.05% (1.50–8.61%) in 50–64 years and 3.45% (0.42–11.91%) in 18–49 years groups (Fig. 3d). Similar proportions of patients with disease progression were observed in patients with eGFR < 30 mL/min/1.73 m^2^ and by COVID-19 severity and age group (Supplementary Fig. S2a–c).

### Recovery

The recovery rate (95% CI) was 72.14% (68.99–75.13%, 611/847) at 28 days which increased to 80.17% (77.32–82.80%) at 56 days in patients with severe CKD (Table 3). It was 55.32% (47.91–62.56%) in CKD stage 4 and 76.93% (73.53–80.10%) in CKD stage 5 at 28 days (Table 3). At 28 days, the recovery rate was 84.40% (80.65–87.68%) in patients with moderate I, 62.40% (57.28–67.32%) in patients with moderate II, and 25.00% (12.12–42.20%) in patients with severe COVID-19. A high recovery rate was observed in younger patients < 65 years (aged 18–49 years: 89.66%, 52/58; aged 50–64 years: 89.86%, 133/148; aged ≥ 65 years: 66.46%, 426/641) (Table 3). Similar results for recovery were observed in patients with eGFR < 30 mL/min/1.73 m^2^ and by COVID-19 severity and age group (Supplementary Table S4).

**Table 3:** Recovery from disease at 28 and 56 days in severe CKD patients and by COVID-19 severity at index and age group

	*N*	28 days		56 days	
		Patients recovered, n	% (95% CI)	Patients recovered, n	% (95% CI)
Severe CKD	847	611	72.14 (68.99–75.13)	679	80.17 (77.32–82.80)
CKD stage 4	188	104	55.32 (47.91–62.56)	136	72.34 (65.36–78.60)
CKD stage 5	659	507	76.93 (73.53–80.10)	543	82.40 (79.27–85.23)
COVID-19 severity at index					
Moderate I	436	368	84.40 (80.65–87.68)	397	91.06 (87.97–93.56)
Moderate II	375	234	62.40 (57.28–67.32)	268	71.47 (66.61–75.99)
Severe	36	9	25.00 (12.12–42.20)	14	38.89 (23.14–56.54)
Age group (years)					
18–49	58	52	89.66 (78.83–96.11)	54	93.10 (83.27–98.09)
50–64	148	133	89.86 (83.83–94.22)	135	91.22 (85.45–95.24)
≥ 65	641	426	66.46 (62.66–70.11)	490	76.44 (72.96–79.68)

% calculated using *N* as denominator

Recovery from COVID-19 defined as patients with healed/cure as reason for discharge

Subgroups of CKD stage 4 defined as those with ICD-10 code N184 or 15 ≤ eGFR < 30 mL/min/1.73 m^2^, excluding those who met the criteria for CKD stage 5; and CKD stage 5 defined as those with ICD-10 code N185 or eGFR < 15 mL/min/1.73 m^2^ or dialysis at index

COVID-19 severity defined as *moderate I,* patients with a record of Emergency Medical Management for moderate COVID-19 and not meeting the criteria for moderate II or severe; *moderate II,* patients requiring non-invasive positive pressure ventilation, high flow oxygen or low flow oxygen; and *severe,* patients requiring invasive mechanical ventilation/extracorporeal membrane oxygenation, or intensive care unit hospitalization

*CI*, confidence interval; *CKD*, chronic kidney disease; *COVID-19*, coronavirus disease-2019; *eGFR*, estimated glomerular filtration rate; *ICD-10,* International Classification of Diseases, 10th revision

## Discussion

This study demonstrated the patient characteristics, treatment pattern, and clinical outcomes in patients with severe CKD, hospitalized with COVID-19 and treated with RDV in Japan. The majority of patients were aged ≥ 65 years, with nearly 45% needing oxygen support at index and with end-stage CKD. The majority of patients recovered and were discharged by 28 days after RDV treatment.

In our study, RDV was initiated within 2 days of hospital admission and treatment duration was 3–5 days, irrespective of CKD stage. A 3-day course of RDV was approved in Japan in March 2022. A survey by the Japanese Society for Dialysis Therapy reported 5–10 days of RDV administration in COVID-19 patients receiving dialysis therapy [23]. At this time, the Omicron variant was the dominant strain and > 80% of people had been vaccinated [24]. Furthermore, these findings align with the previous U.S. study of 42,473 COVID-19 patients, which showed RDV treatment initiation within 2 days of hospital admission and 5-day RDV treatment duration [25].

This study demonstrated inpatient all-cause mortality rate of 11.45% by 28 days and 12.28% by 56 days. In subgroup analysis by CKD stage, COVID-19 severity, and age group, the 95% CIs overlapped for 28-day mortality and disease progression in CKD stages 4 and 5, moderate and severe COVID-19, and age groups 18–49 and 50–65 years. For patients with mild COVID-19 or aged ≥ 65 years, the 95% CIs did not overlap compared to other groups, suggesting higher 28-day all-cause mortality in patients aged ≥ 65 years or with severe COVID-19 disease. A similar trend was evident in patients with eGFR < 30 mL/min/1.73 m^2^. Furthermore, in an international, randomized, placebo-controlled study (the REDPINE study), 29-day all-cause death was 25% in RDV-treated patients with eGFR < 30 mL/min/1.73 m^2^ [19]. In our study, the proportion of death in severe CKD patients was 11.45% (97/847) in inpatient settings. Direct comparison of these results to those of previous studies is limited due to study design and population differences.

Few studies have examined Japanese inpatients with severe CKD and COVID-19. Nevertheless, the findings of this study align with national-level reports from Japan. The COVID‐19 Task Force Committee (May 2020) reported a higher mortality rate in patients undergoing dialysis (16.2%) than the general population (5.3%), and an even higher rate in the subgroup aged ≥ 80 years (30.4% vs 18.9%) [10]. Moreover, the proportion of deaths in this study (12.28%) was consistent with that in the Japanese post-marketing surveillance study (8.98%), and recovery rates in patients with severe CKD (eGFR < 30 mL/min/1.73 m^2^) were better in this study (75.10%) compared to the surveillance population with severe renal impairment (43.75%) [26]. The proportion of deaths in this study is consistent with a Japanese single-center retrospective study showing 13.0% mortality at day 30 post-RDV administration in patients with eGFR < 30 mL/min/1.73 m^2^ [20]. However, a direct comparison is not advisable due to possible differences in vaccination coverage, epidemic strains, and patient characteristics.

Overall, this study, with a large, nationally representative population, provided data on severe CKD patients hospitalized for COVID-19 and treated with RDV primarily during Japan’s Omicron variant epidemic. This study adds to the limited data on CKD patients with COVID-19, a population often excluded from clinical studies. Moreover, findings showed high comorbidity rates and demonstrated that a high proportion of patients without oxygen support recovered with RDV treatment, suggesting the effectiveness of RDV use in early-stage COVID-19 and the importance of timely management in this high-risk population. While defining severe CKD by diagnosis codes in claims data presents potential limitations, the findings were consistent across groups defined by laboratory data.

This study has some limitations, mostly intrinsic to the study design or the database characteristics. The MDV database lacks traceability once patients are discharged or transferred to a different hospital. Hence, patients’ post-discharge outcomes cannot be evaluated. Moreover, diagnosis or treatment from other medical facilities were unknown, and the onset date or initial COVID-19 diagnosis could not be determined. There was also no record for symptom onset timing or cause of death. This was a retrospective, observational, single-arm cohort study using anonymized data of Japanese patients; however, the study design was appropriate to meet the scope of research. The database lacks safety information: hence, the study did not include safety evaluation.

## Conclusion

This study demonstrated that among patients with severe CKD and hospitalized for COVID-19, the majority were aged ≥ 65 years, with moderate to severe COVID-19, about half required oxygen support, and had end-stage CKD. However, most patients recovered by 28 days. This study provides insights into RDV treatment in COVID-19 patients with severe CKD in Japan, which could contribute to discussion on standard of care for patients with severe CKD.

## Supplementary Information

Below is the link to the electronic supplementary material.

Supplementary file1 (DOCX 777 KB)

## Data Availability

The datasets generated and/or analyzed during the current study are not publicly available because the data were obtained from Medical Data Vision, Co., Ltd., Japan.
