# Supplementary material for: Treatment pattern and clinical outcomes of remdesivir in hospitalized COVID-19 patients with severe chronic kidney disease: a database analysis of acute care hospitals in Japan
Source: Clin Exp Nephrol. 2024 Dec 30;29(5):624–37. doi: 10.1007/s10157-024-02609-0 (PMC12049339; doi:10.1007/s10157-024-02609-0)
Supplement: Supplementary file 1 — Supplementary file1 (DOCX 777 KB) [file 10157_2024_2609_MOESM1_ESM.docx]

**Supplementary material**

**Journal:** Clinical and Experimental Nephrology

**Title**

Treatment pattern and clinical outcomes of remdesivir in hospitalized COVID-19 patients with severe chronic kidney disease: a database analysis of acute care hospitals in Japan

**Authors**

Manami Yoshida^1^, Nao Taguchi^1^, Yi Piao^1^, Rikisha Gupta^2^, Mark Berry^2^, Jami Peters^2^, Mazin Abdelghany^2^, Mel Chiang^3^, Chen-Yu Wang^3^, Hiroshi Yotsuyanagi^4^

**Affiliations**

^1^Gilead Sciences, K.K., 16/F GRAN TOKYO SOUTH TOWER, 1-9-2, Marunouchi, Chiyoda-ku, Tokyo, 100-6616, Japan.

^2^Gilead Sciences, Inc., 333 Lakeside Dr, Foster City, CA, USA.

^3^Gilead Sciences Hong Kong Limited, Taiwan Branch.

^4^The Institute of Medical Science, The University of Tokyo, Tokyo, Japan.

**Corresponding author**:

Manami Yoshida

Gilead Sciences, K.K., 16/F GRAN TOKYO SOUTH TOWER, 1-9-2, Marunouchi, Chiyoda-ku, Tokyo, 100-6616, Japan

Contact number: +81-3-6705-1607

Email: Manami.Yoshida3@gilead.com

**Supplementary Table 1** Codes for CKD stage 4/5 and dialysis

|  | Code List |
| --- | --- |
| Diagnosis of CKD stage 4 and 5 | ICD-10 code: N184, N185 |
| Dialysis | Medical procedure code: B001, C102, C102-2, J038, J042 |

*CKD,* chronic kidney disease; *ICD-10*, International Classification of Diseases, 10th revision

**Supplementary Table 2** Demographic and baseline clinical characteristics of patients with eGFR < 30 mL/min/1.73m^2^

| Characteristics | Severe CKD patients with eGFR < 30 mL/min/1.73m^2^  (n = 253) |
| --- | --- |
| Age (years) | |
| Mean ± SD | 77.1 ± 13.6 |
| Age groups (years) | |
| 18−49 | 13 (5.14) |
| 50−64 | 27 (10.67) |
| ≥ 65 | 213 (84.19) |
| Sex | |
| Male | 159 (62.85) |
| Female | 94 (37.15) |
| Comorbidities | |
| COPD | 32 (12.65) |
| Cardiovascular disease | 233 (92.09) |
| Hypertension | 202 (79.84) |
| Diabetes mellitus | 141 (55.73) |
| ESCKD | |
| Dialysis | 73 (28.85) |
| CKD stage 5 | 82 (32.41) |
| eGFR < 15 mL/min/1.73m^2^ | 117 (46.25) |
| COVID-19 severity at index | |
| Moderate I | 122 (48.22) |
| Moderate II | 125 (49.41) |
| Severe | 6 (2.37) |

All data are presented as n (%) unless otherwise specified.

COVID-19 severity defined as *moderate I,* patients with a record of Emergency Medical Management for moderate COVID-19 and not meeting the criteria for moderate II or severe; *moderate II,* patients requiring non-invasive positive pressure ventilation, high flow oxygen or low flow oxygen; and *severe,* patients requiring invasive mechanical ventilation/extracorporeal membrane oxygenation, or intensive care unit hospitalization.

*CKD*, chronic kidney disease; *COPD*, chronic obstructive pulmonary disease; *COVID-19,* coronavirus disease-2019; *eGFR,* estimated glomerular filtration rate; *ESCKD*, end-stage chronic kidney disease; *SD*, standard deviation

**Supplementary Table 3** Treatment pattern of remdesivir in patients with eGFR < 30 mL/min/1.73m^2^

| Treatment pattern | Severe CKD patients with eGFR < 30 mL/min/1.73m^2^ |
| --- | --- |
| Duration of remdesivir treatment (days) (n = 253) | |
| Mean ± SD | 4.5 ± 2.3 |
| Median (Q1−Q3) | 5.0 (3.0−5.0) |
| Time to remdesivir initiation from hospital admission (days) (n = 213) | |
| Mean ± SD | 2.2 ± 2.6 |
| Median (Q1−Q3) | 1.0 (1.0−2.0) |
| Duration of ICU admission^a^ (days) (n = 4) | |
| Mean ± SD | 6.3 ± 5.4 |
| Median (Q1−Q3) | 5.5 (2.0−10.5) |
| Concomitant drugs^b^ (n = 253), n (%) | |
| Corticosteroid | 104 (41.11) |
| Baricitinib | 14 (5.53) |
| Tocilizumab | 5 (1.98) |
| Heparin | 106 (41.90) |

All data are presented as mean ± SD unless otherwise specified.

^a^Including patients who were admitted to the ICU during the follow-up period.

^b^Includes data pertaining to COVID-19 related drugs.

*CKD*, chronic kidney disease; *COVID-19,* coronavirus disease-2019; *eGFR*, estimated glomerular filtration rate; *ICU*, intensive care unit; *Min*, minimum; *Max*, maximum; *Q1*, first quartile; *Q3*, third quartile; *SD*, standard deviation

**Supplementary Table 4** Recovery from disease at 28 and 56 days in patients with eGFR < 30 mL/min/1.73m^2^ by COVID-19 severity and age group

| Recovery from disease |  | 28 days | | 56 days | |
| --- | --- | --- | --- | --- | --- |
|  | N | Patients recovered, n | % (95% CI) | Patients recovered, n | % (95% CI) |
| eGFR < 30 mL/min/1.73m^2^ | 253 | 160 | 63.24 (56.97−69.19) | 190 | 75.10 (69.30−80.30) |
| COVID-19 severity at index | | | | | |
| Moderate I | 122 | 93 | 76.23 (67.68−83.47) | 105 | 86.07 (78.63−91.67) |
| Moderate II | 125 | 65 | 52.00 (42.89−61.02) | 83 | 66.40 (57.40−74.60) |
| Severe | 6 | 2 | 33.33 (4.33−77.72) | 2 | 33.33 (4.33−77.72) |
| Age group (years) | | | | | |
| 18−49 | 13 | 12 | 92.31 (63.97−99.81) | 13 | 100.00 (75.29−100.00) |
| 50−64 | 27 | 23 | 85.19 (66.27−95.81) | 23 | 85.19 (66.27−95.81) |
| ≥ 65 | 213 | 125 | 58.69 (51.76−65.37) | 154 | 72.30 (65.77−78.20) |

Recovery from COVID-19 defined as patients with healed/cure as reason for discharge.

COVID-19 severity defined as *moderate I,* patients with a record of Emergency Medical Management for moderate COVID-19 and not meeting the criteria for moderate II or severe; *moderate II,* patients requiring non-invasive positive pressure ventilation, high flow oxygen or low flow oxygen; and *severe,* patients requiring invasive mechanical ventilation/extracorporeal membrane oxygenation, or intensive care unit hospitalization.

*CI*, confidence interval; *CKD*, chronic kidney disease; *COVID-19*; coronavirus disease-2019; *eGFR*, estimated glomerular filtration rate

**Supplementary Fig. 1** Kaplan-Meier curve for inpatient all-cause mortality over 56 days in **(a)** patients with eGFR < 30 mL/min/1.73m^2^, **(b)** patients with eGFR < 30 mL/min/1.73m^2^ based on COVID-19 severity at index, **(c)** patients with eGFR < 30 mL/min/1.73m^2^ based on age group


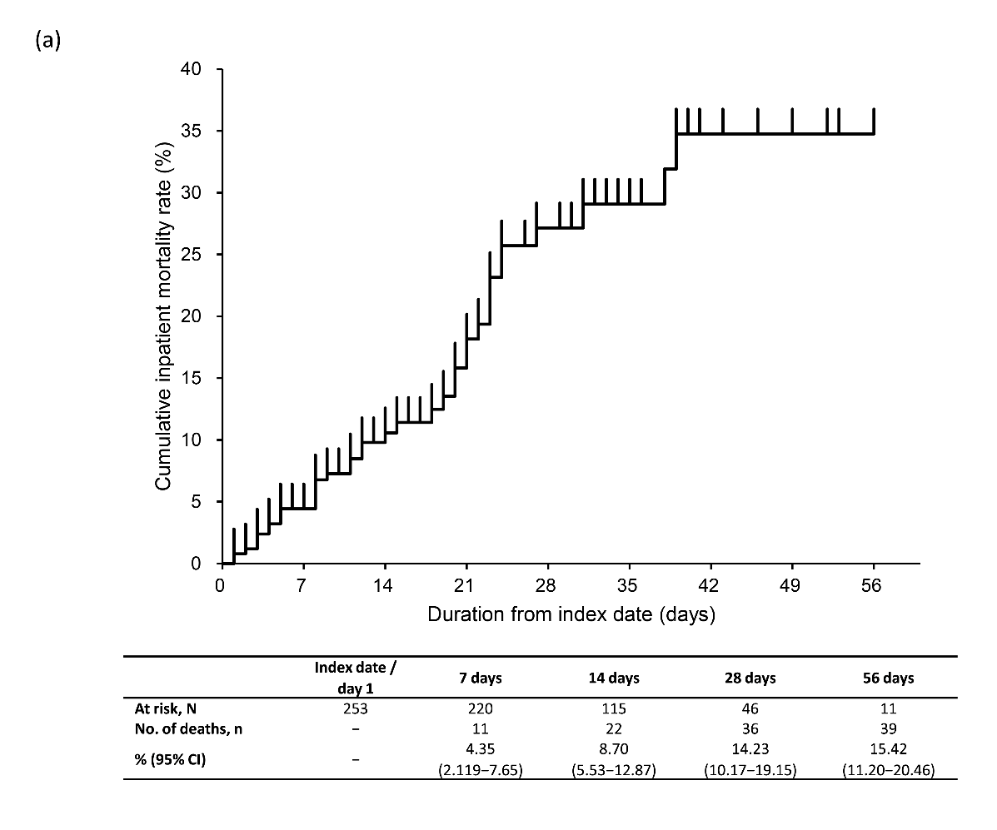


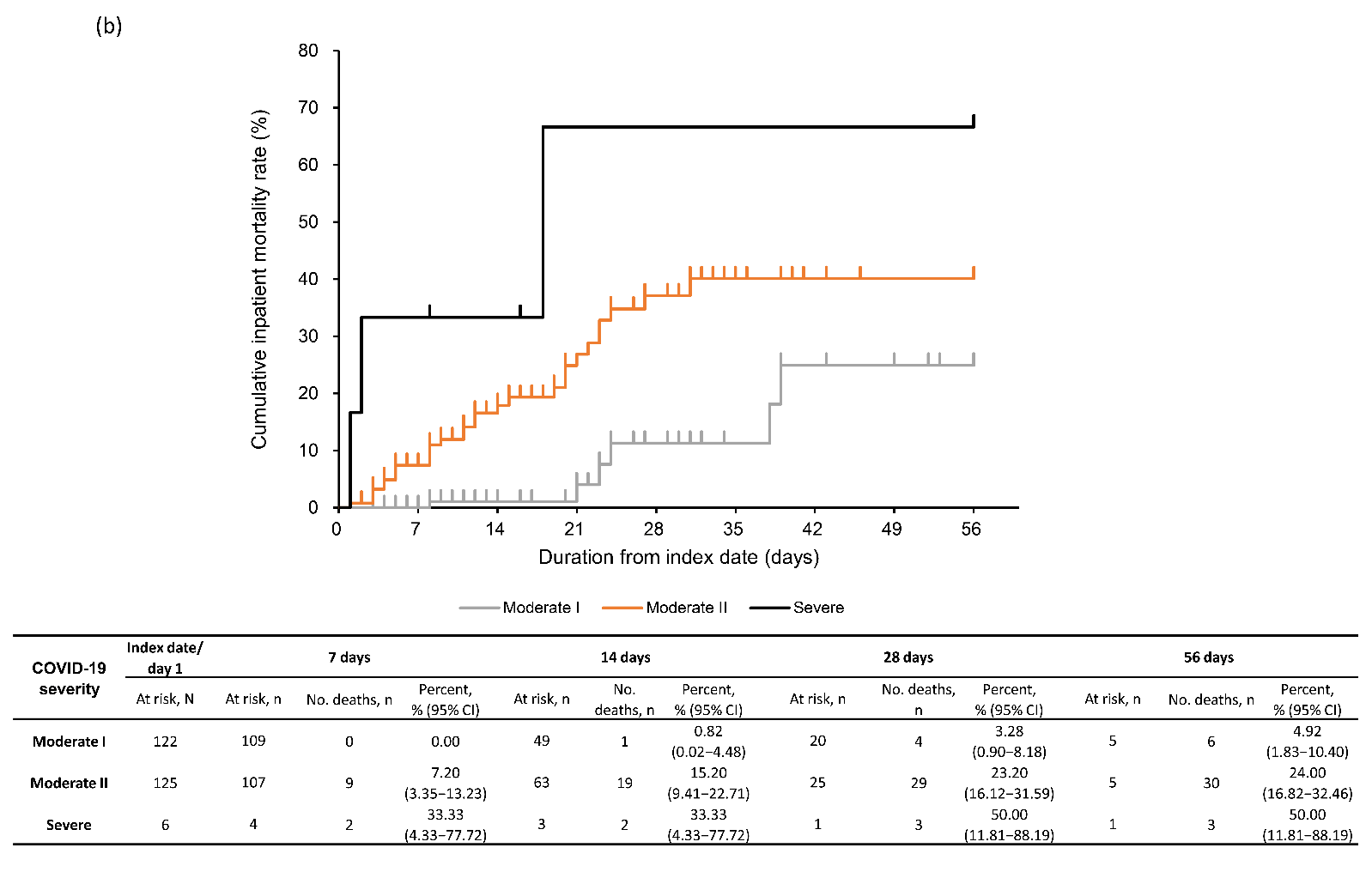


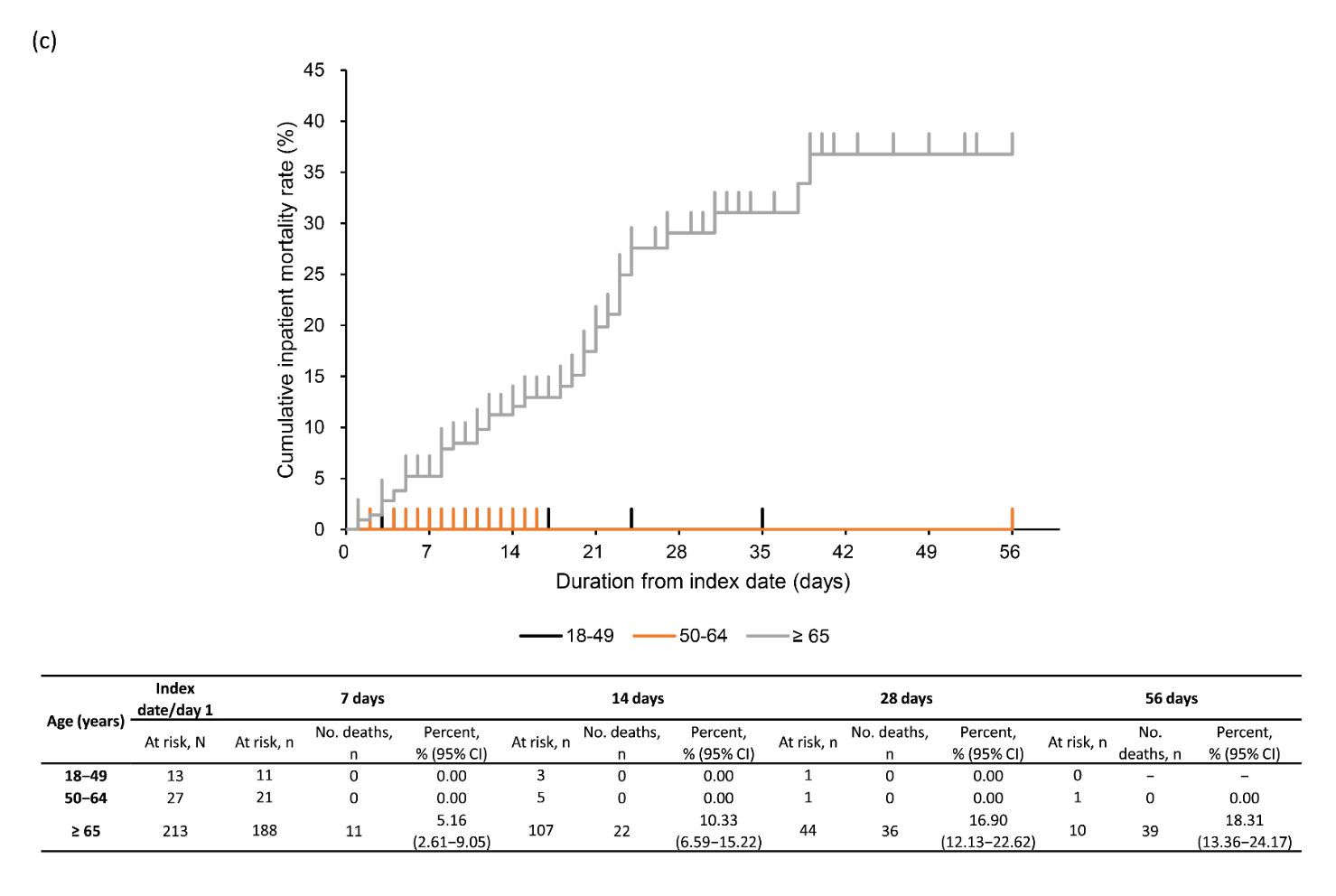


COVID-19 severity defined as *moderate I,* patients with a record of Emergency Medical Management for moderate COVID-19 and not meeting the criteria for moderate II or severe; *moderate II,* patients requiring non-invasive positive pressure ventilation, high flow oxygen or low flow oxygen; and *severe,* patients requiring invasive mechanical ventilation/extracorporeal membrane oxygenation, or intensive care unit hospitalization.

*CI*, confidence interval; *CKD*, chronic kidney disease; *COVID-19,* coronavirus disease-2019; *eGFR*, estimated glomerular filtration rate; *No.*, number

**Supplementary Fig. 2** Kaplan-Meier curve for disease progression over 56 days in **(a)** patients with eGFR < 30 mL/min/1.73m^2^, **(b)** patients with eGFR < 30 mL/min/1.73m^2^ based on COVID-19 severity at index, **(c)** patients with eGFR < 30 mL/min/1.73m^2^ based on age group


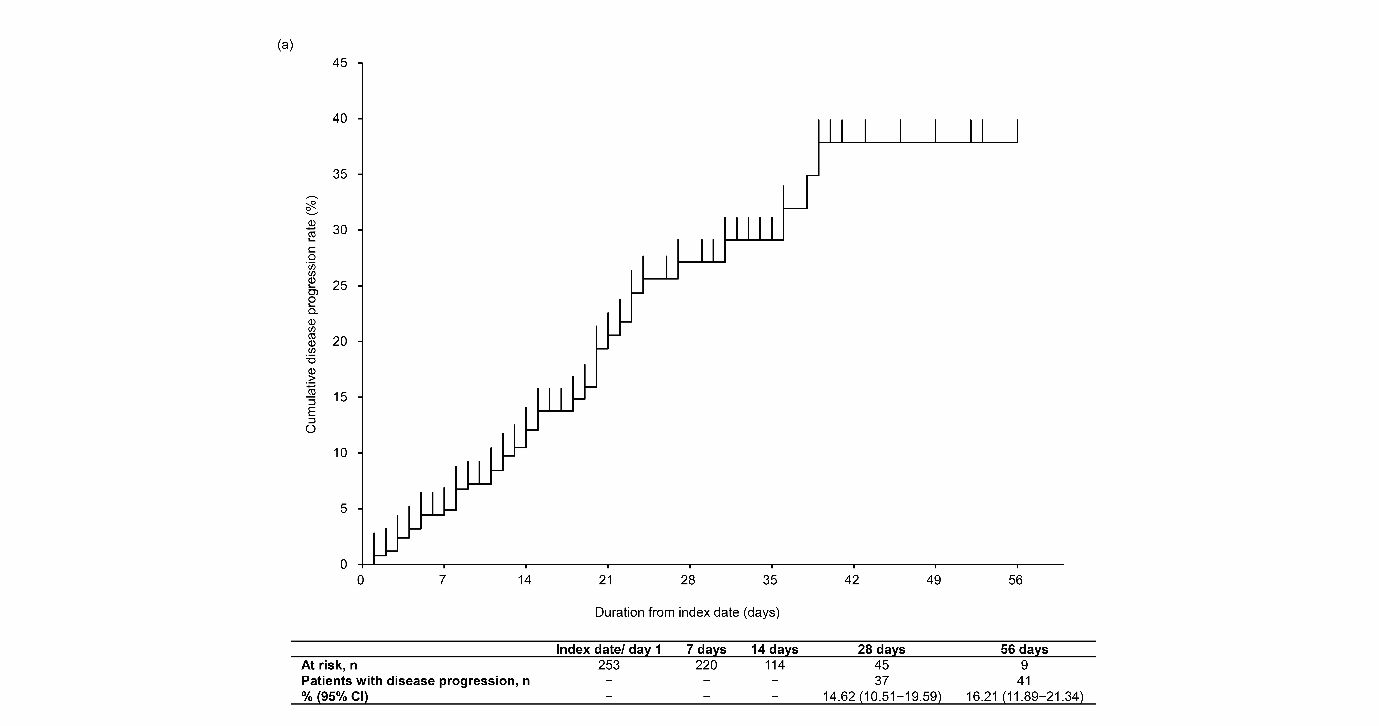


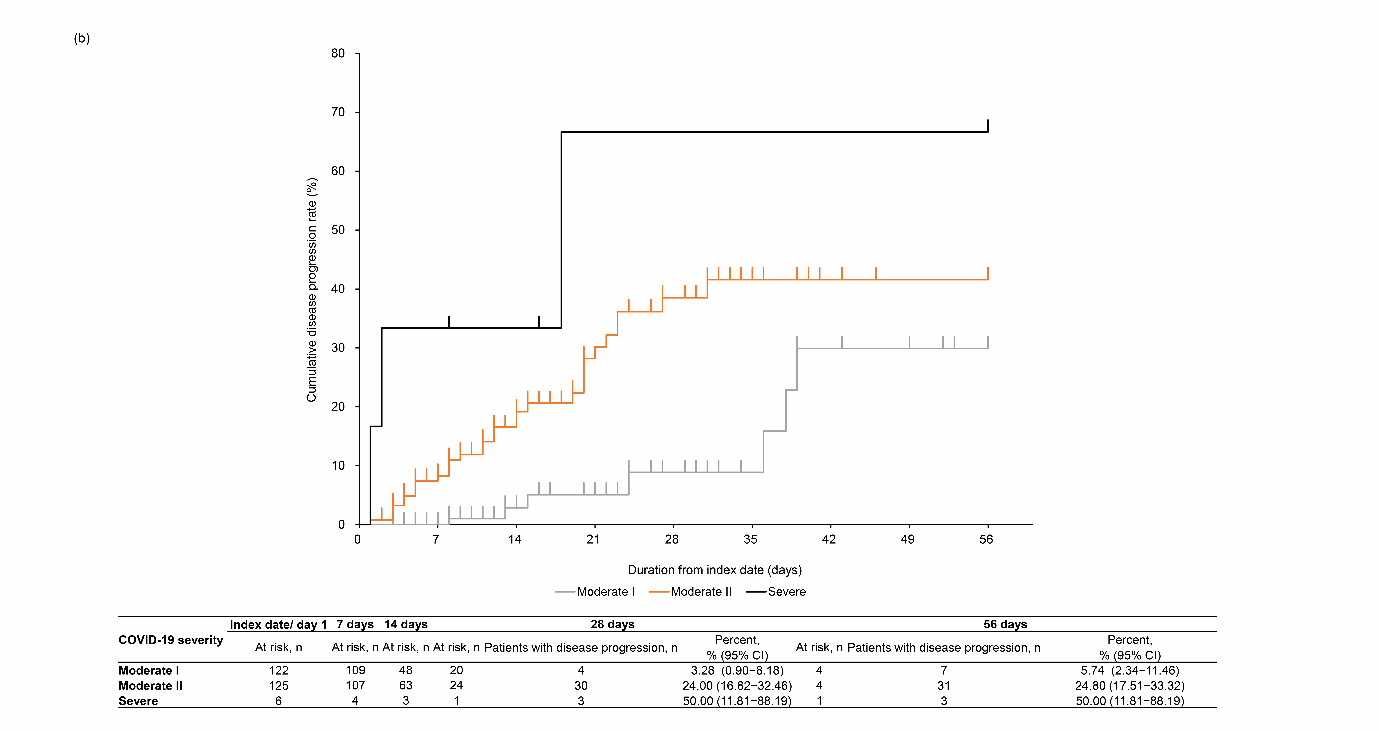


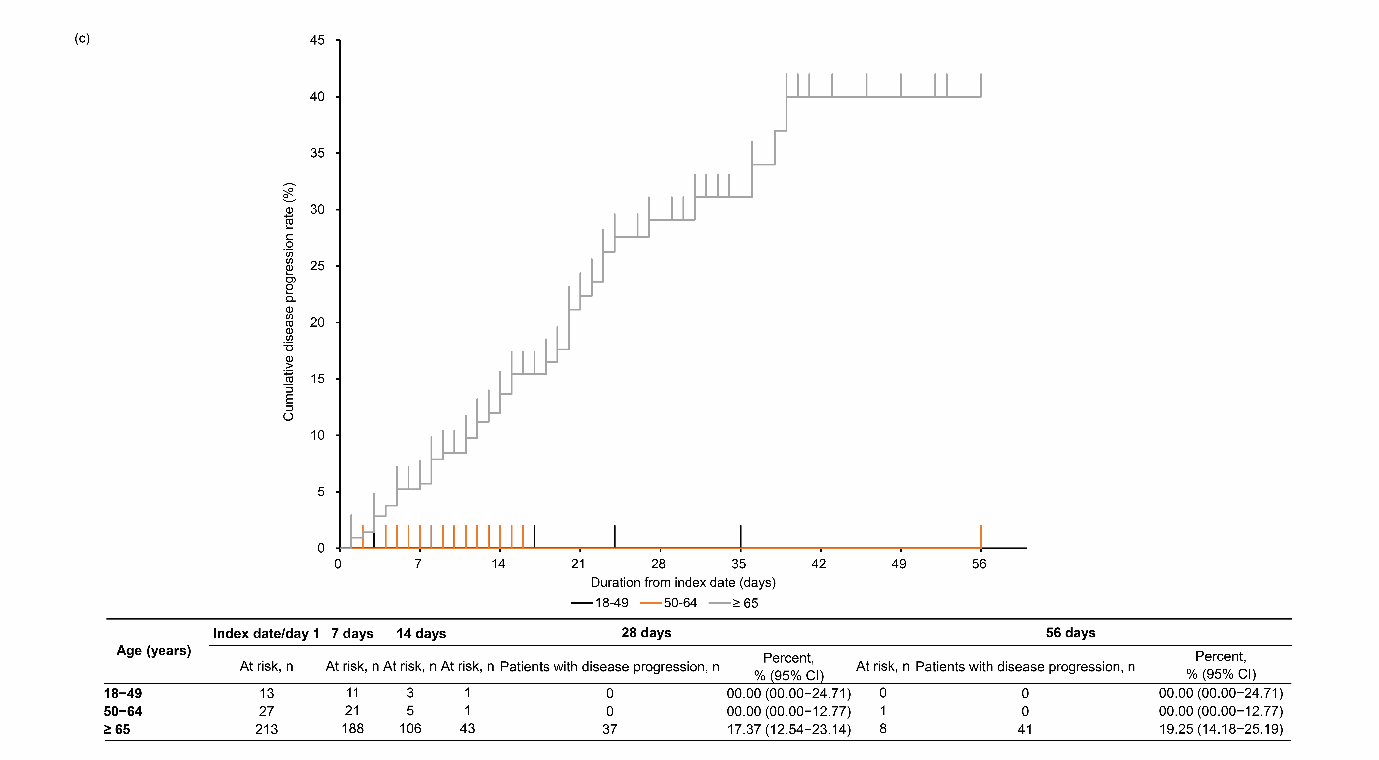


Number and percentage of patients with disease progression estimated at 28 and 56 days.

Disease progression in patients with moderate I or II COVID-19 at index, defined as having a record for invasive mechanical ventilation/extracorporeal membrane oxygenation, or intensive care unit hospitalization, or as death during follow-up; and in patients with severe COVID-19 at index, defined as death during follow-up.

COVID-19 severity defined as *moderate I,* patients with a record of Emergency Medical Management for moderate COVID-19 and not meeting the criteria for moderate II or severe; *moderate II,* patients requiring non-invasive positive pressure ventilation, high flow oxygen or low flow oxygen; and *severe,* patients requiring invasive mechanical ventilation/extracorporeal membrane oxygenation, or intensive care unit hospitalization.

*CI*, confidence interval; *CKD*, chronic kidney disease; *COVID-19,* coronavirus disease-2019; *eGFR*, estimated glomerular filtration rate
